# Changes in functional outcome over five years after stroke

**DOI:** 10.1002/brb3.1300

**Published:** 2019-05-07

**Authors:** Åsa Rejnö, Salmir Nasic, Kerstin Bjälkefur, Eric Bertholds, Katarina Jood

**Affiliations:** ^1^ Department of Medicine Skaraborg Hospital Skövde Skövde Sweden; ^2^ Department of Health Sciences University West Trollhättan Sweden; ^3^ Research and Development Centre Skaraborg Hospital Skövde Skövde Sweden; ^4^ Department of Health and Social Care Municipality of Lidköping Sweden; ^5^ Institute of Neuroscience and Physiology, Department of Clinical Neuroscience The Sahlgrenska Academy at University of Gothenburg Gothenburg Sweden

**Keywords:** functional outcome, quality register, longitudinal study, stroke

## Abstract

**Objectives:**

Data on the long‐term time course of poststroke functional outcome is limited. We investigated changes in functional outcome over 5 years after stroke in a hospital based cohort.

**Materials and Methods:**

Consecutive patients who were independent in activities of daily living (ADL) and admitted to a Stroke Unit at Skaraborg Hospital, Sweden for a first acute stroke from 2007 to 2009 (*n* = 1,421) were followed‐up after 3 months and thereafter annually over 5 years using a postal questionnaire. Clinical variables at acute stroke and 3 months post stroke were obtained from the Swedish Stroke Register. ADL dependency was defined as dependence in dressing, toileting or indoor mobility.

**Results:**

The proportions of survivors who reported ADL dependency remained stable throughout follow‐up (19%–22%). However, among survivors who were ADL independent at 3 months, about 3% deteriorated to dependency each year. Deterioration was predicted by age (HR 1.11; 95% CI 1.08–1.13), diabetes (HR 1.65; 95% CI 1.12–2.44), NIHSS score (HR 1.07; 95% CI 1.04–1.10), and self‐perceived unmet care needs one year post stroke (HR 2.01; 95% CI 1.44–2.81). Transitions from ADL dependency to independence occurred mainly during the first year post stroke. Improvement was negatively predicted by living alone before stroke (HR 0.41 95% CI 0.19–0.91), NIHSS score (HR 0.90; 95% CI 0.86–0.95) and ischemic stroke (vs. hemorrhagic stroke), HR 0.39; 95% CI 0.17–0.89.

**Conclusion:**

Transitions between ADL independence and dependency occur up to 5 years after stroke. Some of the factors predicting these transitions are potentially modifiable.

## 1 INTRODUCTION

Stroke survivors frequently suffer disabilities that hamper their everyday life and make them dependent on others. Given that the absolute numbers of stroke survivors are increasing, a consequence of aging and population growth in combination with improved stroke care (Wolfe et al., 2011), the burden on the society from the long‐term effects of stroke is expected to increase.

Despite this, knowledge about the long‐term course of stroke disability is limited. So far, few large‐scale longitudinal long‐term studies with repeated measures of functional outcomes after stroke have been reported (Dhamoon et al., 2009; Luengo‐Fernandez et al., 2013; Ullberg, Zia, Petersson, & Norrving, 2015; Wolfe et al., 2011). Some of these report relatively stable levels of disability with respect to dependency in activities of daily living (ADL) over 5–10 years after stroke (Luengo‐Fernandez et al., 2013; Wolfe et al., 2011). Others report increasing (Dhamoon et al., 2009; Ullberg et al., 2015) or decreasing (de Campos et al., 2017) ADL dependence beyond the first three months after stroke, thus challenging the concept that the chronic phase of stroke is stable (Kwakkel & Kollen, 2013).

The gap of knowledge with respect to the long‐term course of functional outcomes after stroke is an obstacle for delivering adequate care and support to stroke survivors. If disability during the chronic state after stroke is not stable, it may also be modifiable. Thus, better knowledge of the long‐term course of disability after stroke is crucial for the development of timely interventions that can better meet the needs of people afflicted by stroke.

The aim of this study was to contribute descriptive data on the time course of disability over 5 years after stroke, and to identify predictors for improvement as well as deterioration beyond the first three months after stroke.

## 2 MATERIALS AND METHODS

### 2.1 Materials

Data were obtained from two quality registers assessing stroke care; the Swedish Stroke Register (Riksstroke) (Asplund et al., 2011), and the Skaraborg Longitudinal Stroke Register (SLAG). The SLAG register is a local register containing data from annual follow‐ups over 5 years for all patients with an acute stroke treated at the Stroke Units at the Skaraborg Hospitals. The register was established in collaboration with the Swedish Stroke Register in order to assess quality aspects of long‐term stroke care. The Skaraborg Hospital has two collaborating Stroke Units located at two county hospitals, serving approximately 285,000 inhabitants of the entire Skaraborg County in southwestern Sweden. According to local and national guidelines, all patients presenting with a suspected acute stroke, regardless of severity and age, occurring in the Skaraborg County should be admitted to one of the two Stroke Units at the Skaraborg Hospital. Assessments from the Swedish Stroke Register (Riks‐Stroke, 2011) show that a high proportion (>90%) of acute stroke patients were cared for at Stroke Units at the Skaraborg Hospitals during the study period. For the present study, we included all patients who were independent in ADL before stroke and were admitted to one of the Stroke Units at the Skaraborg Hospitals with a first ever stroke between 1 January 2007 and 31 December 2009.

### 2.2 Ethical considerations

Written information about the SLAG register, the voluntary nature of participation, and the possibility to actively decline participation was given to the participants during their hospital stay. As data was collected primarily for quality assessment, formal written informed consent was not obtained. Approval to use the collected data in the quality register for a retrospective analysis was received from The Regional Ethics Board in Gothenburg (ref.nr 270‐14).

### 2.3 Clinical variables at baseline and at three months post stroke

Data on age at acute stroke, sex, previous strokes, ability in ADL before and 3 months post stroke, living situation before and post stroke, type of stroke, stroke severity at admission measured as National Institute of Health Stroke Scale (NIHSS) score, presence of vascular risk factors, and secondary preventive treatments at discharge were obtained from the Swedish Stroke Register. In this register ability in ADL 3 months post stroke is assessed by three questions with the following response alternatives; (a) “How is your mobility now? Independent/independent indoors/need help”, (b) “Do you need help from someone to visit the toilet? Yes/No,” and (c) “Do you need help getting dressed and undressed? Yes/ No” (Eriksson, Appelros, Norrving, Terént, & Stegmayr, 2007). ADL dependency was defined as needing help with indoor mobility, dressing or toileting, whereas those who were independent in indoor mobility, dressing and toileting were regarded as ADL independent.

### 2.4 Clinical variables one to five years post stroke

Data were collected by postal questionnaires sent annually to all stroke survivors one to five years post stroke. Information on vital status was obtained by linkage to the Swedish Population Register. The annual postal questionnaire was developed in collaboration with the Swedish Stroke Register. In order to enable comparison with functional outcome at 3 months, the postal questionnaire was based on the Swedish Stroke Register's survey for three‐month follow‐up of stroke survivors and included the identical questions and response alternatives about ADL. Both questionnaires included instructions to the respondents to use help from a relative or a caregiver if they were unable to complete the questionnaire on their own. The annual follow‐up questionnaires also included questions about self‐perceived unmet care needs. The latter included the following five items; “Have your needs of home care service been met with respect to (a) Health care (described as help with medication, wound dressing or catheter care), (b) Service (described as help with cleaning or grocery shopping), (c) Personal care (described as help with dressing, hygiene, or toileting)?”, “Have your needs of disability aids been met?”, and “Have your needs of rehabilitation or training after stroke been met?”. The response alternatives for the questions about unmet care demands were “No needs”, “Fulfilled needs”, “Partly unmet needs”, “Completely unmet needs” and “Does not know”. Self‐perceived unmet care needs were defined as perceiving one of these items as partly or completely unmet. The other response alternatives were regarded as perceiving their needs for care as fulfilled.

### 2.5 Patients lost to follow‐up

Patients who did not return the questionnaire received one postal reminder and the study nurse made attempts to contact them by telephone. The group “Patients lost to follow‐up” comprised those patients who did not return the questionnaire after one reminder, were not available by telephone and persons who had no valid address. Declining patients were not invited for further follow‐ups.

### 2.6 Statistical analyses

Descriptive statistics as mean and standard deviation for continuous data and frequencies and percentages for categorical data was presented. Change in ADL ability from 3 months to 5 years post stroke was analyzed separately in survivors with respect to ADL ability 3 months post stroke using the Kaplan‐Meier method. For both groups this analysis was stratified with respect to age (up to and above 75 years). Factors associated with improvement or deterioration in ADL ability were investigated separately using univariate and multivariate Cox logistic regression models. Variables associated with improvement or deterioration in ADL ability in univariate Cox regression analyses (*p* < 0.1) were selected for the multivariate models. As patients did not report on their perception of met or unmet care needs at 3 months follow‐up, we used data from follow‐up at one year after stroke for this variable. To investigate to what extent the results were influenced by those who deteriorated or improved between three months and one year, i.e., before the perception of met or unmet care needs were collected, a sensitivity analysis excluding individuals deteriorating from ADL independency to dependency between three months and one year was performed. *p*‐values <0.05 were considered as statistical significant. IBM SPSS v.22 was used to perform all statistical analysis.

## 3 RESULTS

**Figure 1:**
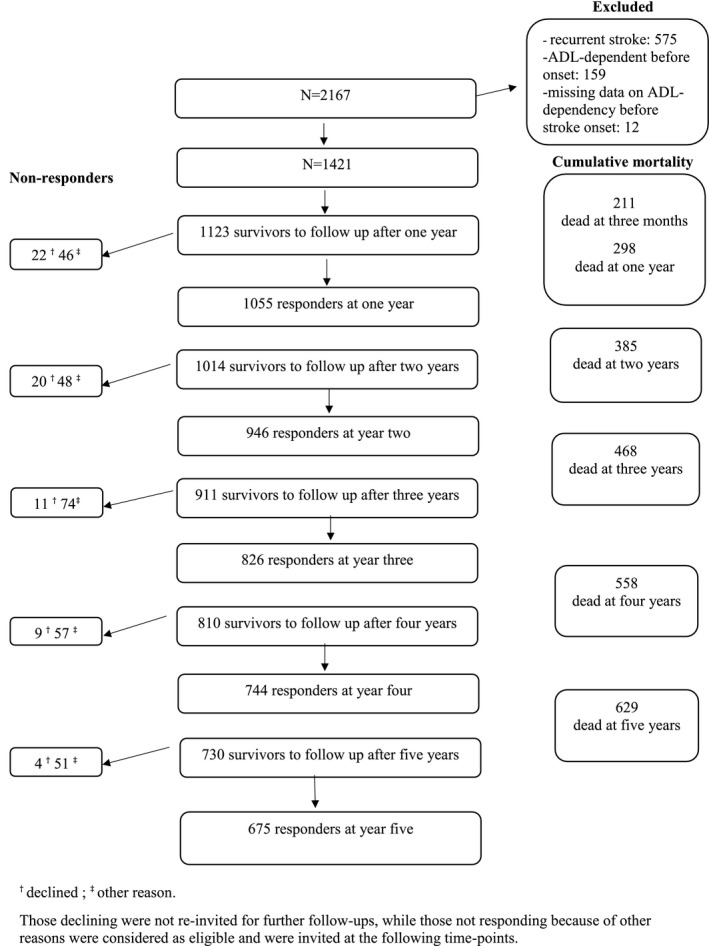
Study flowchart

In total, 2,167 stroke events were recorded during the study period. Of these, 1,421 were first‐ ever strokes in previously ADL‐independent individuals. Baseline characteristics for the 1,421 participants and perceived unmet care needs at the first annual follow‐up are given in Table 1. The mean age was 75.9 years, 643 (45%) were females, mean NIHSS was 6.4, and 90% of the strokes were ischemic. At one year after stroke 324 (31%) reported self‐perceived unmet care needs. The most common reported unmet care need were rehabilitation, reported by 22%, followed by disability aids (10%), service (8%), health care (6%) and personal care (5%). Missing data was low for all variables (<8%), except for the NIHSS score for which data was missing in 22% at baseline and in 18% among survivors at 3 months post stroke. A study flow chart showing the follow‐up annually over the 5 years is given in Figure 1. At each time point, the response rate was >90%. The proportion of the returned questionnaires that were answered by the stroke survivors themselves was stable during follow‐up and between 57%–63% at the different time points. The corresponding proportions of returned questionnaires answered by the stroke survivor with help from a relative or care giver was 24%–31%, and 11%–13% for questionnaires completely answered by relatives or care givers. Among those returning the questionnaires, information about ADL status was missing in 5%, 7%, 9%, 7% and 8% at year one, two, three, four and five, respectively.

**Table 1:** Characteristics of the study population

	All (*n* = 1,421)	<75 years (*n* = 563)	≥75 years (*n* = 858)
Sex			
Females, *n* (%)	643 (45)	197 (35)	446 (52)
Type of stroke			
Ischemic stroke, *n* (%)	1,285 (90)	491 (87)	794 (93)
Hemorrhagic stroke, *n* (%)	126 (9)	68 (12)	58 (7)
Undetermined, *n* (%)	9 (1)	4 (1)	5 (1)
NIHSS total score, mean (*SD*)^a^	6.4 (7.6)	5.1 (6.7)	7.4 (8.0)
NIHSS 0–5, *n* (%)	704 (49)	333 (59)	371 (43)
NIHSS 6–14, *n* (%)	237 (17)	78 (14)	159 (18)
NIHSS ≥ 15, *n* (%)	167 (12)	49 (9)	118 (14)
Missing NIHSS‐data, *n* (%)	313 (22)	103 (18)	210 (24)
Living alone before stroke onset, *n* (%)	658 (46)	178 (32)	480 (56)
Use of antihypertensive at stroke onset, *n* (%)	764 (54)	242 (43)	522 (61)
Diabetes, *n* (%)	261 (18)	105 (19)	156 (18)
Current smoker, *n* (%)	172 (12)	130 (24)	42 (5)
Atrial fibrillation, *n* (%)	395 (28)	72 (13)	323 (38)
Housing situation, pre stroke			
Ordinary housing, *n* (%)	1,203 (85)	538 (96)	665 (77)
Ordinary housing with home care, *n* (%)	178 (12)	19 (3)	159 (19)
Assisted living^b^, *n* (%)	40 (3)	6 (1)	34 (4)
Discharged to			
Independent living, *n* (%)	808 (57)	378 (67)	430 (50)
Assisted living^b^, *n* (%)	377 (26)	64 (11)	313 (36)
Geriatric‐/Rehabilitation clinic, *n* (%)	82 (6)	78 (14)	4 (1)
Other^c^, *n* (%)	17 (1)	13 (2)	4 (1)
Deceased, *n* (%)	136 (10)	29 (5)	107 (12)
Medication at discharge			
Antiplatelet, *n* (%)	949 (69)	405 (73)	544 (66)
Antihypertensive, *n* (%)	1,083 (78)	422 (75)	661 (77)
Anticoagulantia (Warfarin), *n* (%)	192 (14)	63 (11)	129 (16)
Statin, *n* (%)	580 (42)	325 (58)	255 (30)
Perceived unmet care needs at one year^d^, *n* (%)	324 (31)	110 (23)	214 (38)

a NIHSS score was available in 1,108 (78%).

b Community nursing home or nursing home with stroke rehabilitation.

c Still hospitalized at other acute clinic or other stroke unit, unknown.

d Patient reported unmet care needs at follow‐up after 1 year.

**Figure 2:**
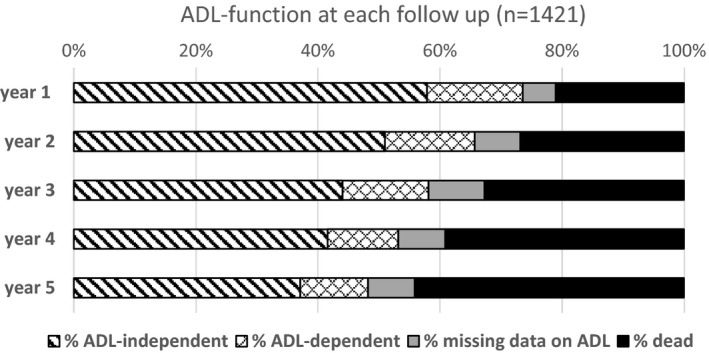
Outcomes at follow‐up with respect to death and dependence/independence in activities of daily living

The cumulative survival was 85% at 3 months, 79% at year one, 72% at year two, 66% at year three, 59% at year four and 54% at year five post stroke. Over the first two years, survival was poorer among those with hemorrhagic stroke with a cumulative survival of 76% at year one, 73% at year two, 65% at year three, 62% at year four, and 57% at year five. At 3 months, 908 survivors reported ADL independency, 272 reported ADL dependency, 211 were dead, and data on ADL status was missing in 30. As shown in Figure 2 the absolute number of ADL‐dependent stroke survivors decreased over time. However, the proportion of survivors reporting ADL dependency remained stable (22% at 3 months, 20% at one, 20% at two, 21% at three, 19% at four, and 20% at five years post stroke).

**Figure 3:**
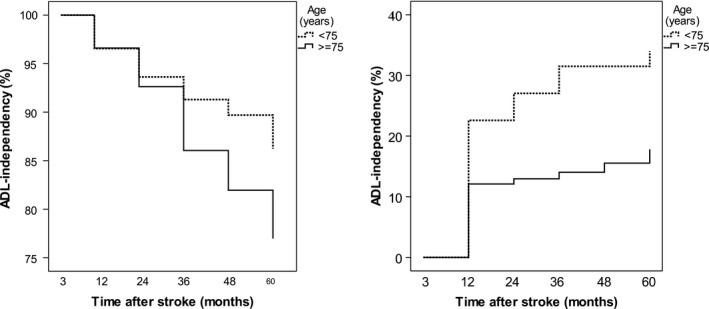
Time to activity in daily life (ADL) dependency (deterioration, a) and ADL independency (improvement, b) according to age, in survivors ADL independent at 3 months (*n* = 908) (a) and in survivors ADL dependent at 3 months (*n* = 272) (b)

As shown in Figure 3, Panel A, ADL‐independent survivors at 3 months after stroke deteriorated to ADL dependency at a slow but constant rate throughout follow‐up. In total 192 individuals deteriorated, with cumulative proportions of 3% at one, 6% at two, 11% at three, 14% at four, and 18% at five years post stroke. Similar patterns were observed in subjects above and less than 75 years of age, although those <75 years deteriorated at a slower rate. Cox regression multivariable analysis identified age (hazard ratio [HR] 1.11, 95% confidence interval [CI] 1.08–1.13), diabetes (HR 1.65; 95% CI 1.12–2.44), NIHSS score (HR 1.07, 95% CI 1.04–1.10), and self‐perceived unmet care needs (HR 2.01; 95% CI 1.44–2.81) as independent predictors for deterioration to ADL dependency (Table 2). Analysis, in which we excluded those who deteriorated to ADL dependence between the three‐month and the one‐year follow‐up, showed similar results (data not shown). Among the 192 individuals censored for a first deterioration during follow‐up, 31 (16%) returned to ADL independency during follow‐up.

**Table 2:** Predictors for deterioration to ADL dependency during follow‐up

Variable	Univariable			Multivariable		
	HR	95% CI	*p*‐value	HR	95% CI	*p*‐value
Age, per year	1.09	1.07–1.11	<0.001	1.11	1.08–1.13	<0.001
Women	1.65	1.24–2.19	0.001	1.32	0.92–1.88	0.130
Smoking	0.84	0.55–1.28	0.427	–	–	–
Living alone before stroke^a^	1.79	1.34–2.38	<0.001	1.06	0.73–1.54	0.765
Diabetes	1.74	1.27–2.41	0.001	1.65	1.12–2.44	0.012
Antihypertensive at admission	1.36	1.02–1.81	0.036	0.90	0.63–1.28	0.898
Ischemic stroke^b^	1.63	0.83–3.18	0.154	–	–	–
NIHSS, per one point increase	1.06	1.03–1.09	<0.001	1.07	1.04–1.10	<0.001
Atrial fibrillation	1.69	1.23–2.32	0.001	0.96	0.65–1.40	0.819
Statin at discharge	0.55	0.41–0.73	<0.001	1.07	0.76–1.51	0.696
Anticoagulation at discharge	1.03	0.71–1.50	0.861	–	–	–
Antihypertensive at discharge	1.11	0.76–1.62	0.591	–	–	–
Antiplatelets at discharge	0.88	0.64–1.21	0.448	–	–	–
Self‐perceived unmet care needs^c^	2.96	2.20–3.98	<0.001	2.01	1.44–2.81	<0.001

Note

Cox regression analysis. Variables associated with deterioration in ADL ability in univariate Cox regression analyses (*p* < 0.1) were selected for the multivariate model.

a Compared to “sharing household”.

b Ischemic stroke compared to hemorrhagic stroke.

c Measured one year after stroke.

Improvement to ADL independency occurred in 44 of those 272 reporting ADL dependency 3 months after stroke. This transition occurred mainly during the first year, but was observed throughout the follow‐up, especially in those <75 years (Figure 3, Panel B). In this group, one third of those improving to ADL independency during follow‐up did so during the second and third year after stroke. Cox regression multivariable analysis (Table 3) identified living alone before stroke (HR 0.41; 95% CI 0.19–0.91), NIHSS score (HR 0.90, 95% CI 0.86–0.95), and ischemic stroke (vs. hemorrhagic stroke), HR 0.39; 95% CI 0.17–0.89. Among the 44 individuals who were censored for a first transition to independency, 24 (54%) remained independent throughout follow‐up.

**Table 3:** Predictors for improvement to ADL independency during follow‐up

Variable	Univariable			Multivariable		
	HR	95% CI	*p*‐value	HR	95% CI	*p*‐value
Age	0.97	0.94–1.00	0.055	0.97	0.94–1.01	0.141
Women	1.28	0.71–2.31	0.419	–	–	–
Smoking	1.55	0.55–4.35	0.402	–	–	–
Living alone before stroke^a^	0.47	0.25–0.90	0.023	0.41	0.19–0.91	0.029
Diabetes	1.25	0.58–2.70	0.563	–	–	–
Antihypertensive at admission	1.29	0.71–2.36	0.404	–	–	–
Ischemic stroke^b^	0.50	0.25–1.01	0.052	0.39	0.17–0.89	0.025
NIHSS, per one point increase	0.91	0.87–0.96	<0.001	0.90	0.86–0.95	<0.001
Atrial fibrillation	0.87	0.46–1.65	0.675	–	–	–
Statin at discharge	1.29	0.70–2.39	0.412	–	–	–
Warfarin at discharge	1.09	0.46–2.58	0.843	–	–	–
Antihypertensive at discharge	2.54	0.91–7.11	0.075	3.06	0.94–10.3	0.065
Antiplatelets at discharge	0.68	0.37–1.24	0.209	–	–	–
Self‐perceived unmet care needs^c^	0.69	0.38–1.27	0.114	–	–	–

Note

Cox regression analysis. Variables associated with deterioration in ADL ability in univariate Cox regression analyses (*p* < 0.1) were selected for the multivariate model.

a Compared to “sharing household”.

b Ischemic stroke compared to hemorrhagic stroke.

c Measured one year after stroke.

## 4 DISCUSSION

Our results show that the proportion of survivors that is dependent on others for ADL remained relatively constant over 5 years post stroke. However, when investigating the trajectories of disability stratified by ADL dependency at 3 months after stroke, we found that transitions between dependency and independency occurred throughout the study period. Deterioration from ADL independence to dependency from 3 months and onwards was predicted by age, NIHSS score at baseline, diabetes and self‐perceived unmet care needs, while improvement from ADL dependency to independence was predicted by living alone before stroke, ischemic stroke, and NIHSS score at baseline, (all inverse associations).

The chronic phase beyond the first year after stroke has so far received little attention. It has been perceived as relatively static, an assumption that is supported by studies showing a strong association between disabilities at 3 months post stroke and long‐term outcomes (Hankey, Jamrozik, Broadhurst, Forbes, & Anderson, 2002; Magalhaes et al., 2014), and large longitudinal cohort studies showing that the proportion of survivors with ADL dependency remains stable over time (Luengo‐Fernandez et al., 2013; Wolfe et al., 2011). However, our results indicate that the relatively stable proportion of ADL dependency at different time points after stroke does not reflect a static course, but rather the balance between death of ADL‐dependent subjects and a net flow of survivors converting from independency to dependency.

Among the survivors who were independent in ADL at 3 months, about 3% per year deteriorated to ADL dependency during follow‐up. Clearly, part of this deterioration could be explained by increasing disability related to aging. However, it is important to note that this deterioration also occurred among younger stroke survivors, not normally subjected to deterioration solely because of increasing age. Moreover, besides age, NIHSS score, diabetes and self‐perceived unmet care needs predicted deterioration to dependency. A similar modest annual decline in ADL function during 5 years of follow‐up was observed in the Northern Manhattan Study (Dhamoon et al., 2009), with a poorer course in those with Medicaid or no health insurance, possibly due to more limited access to rehabilitation and care services. In line with these findings, van de Port, Kwakkel, van Wijk, and Lindeman (2006) reported that deterioration in mobility between 1 to 3 years after stroke was predicted by a poor level of activity. Our observation that stroke survivors' self‐perception of unmet care needs may predict deterioration raises the question if it would be possible to modify this course with delivery of care, support and rehabilitation that meets the stroke survivors' perception of needs. It also calls for further studies exploring stroke survivor's perception of care needs in the chronic phase after stroke.

Also, among those with ADL dependency at 3 months post stroke, the functional status was not static and transitions to ADL independency occurred up to 5 years after stroke, especially in those <75 years of age at acute stroke. Significant improvements of disability in the chronic phase after stroke has been reported before (Hankey et al., 2002; Magalhaes et al., 2014; van de Port et al., 2006), but have so far received little attention. We found that improvement to ADL independence was less likely to occur among those living alone at acute stroke. The underlying mechanisms explaining these associations are not clear, but the observations are in line with studies showing poorer general health and prognosis among those living alone (Reblin & Uchino, 2008; Rendall, Weden, Favreault, & Waldron, 2011; Scheurer, Choudhry, Swanton, Matlin, & Shrank, 2012). However, it should be noted that the relatively low absolute numbers contributing to the analyses of transitions among the ADL‐dependent survivors limited the statistical power. Nevertheless, the observation of a nonstatic long‐term course warrants further studies on how improvements in those with ADL dependency could be supported in the chronic phase after stroke.

Recently, a substantial increase in ADL dependency among stroke survivors between 3 and 12 months after stroke was reported from the Swedish Stroke Register by Ullberg et al. (2015). In that study, the ADL dependency rate among stroke survivors increased from 16.2% at 3 months to 28.3% at 12 months. Deterioration to ADL dependency from 3 to 12 months was predicted by female sex, diabetes, stroke severity, previous stroke, stroke type and atrial fibrillation. Some of the methods used in the study by Ullberg et al. (2015) are similar to our study. Both studies are hospital based, were conducted in Sweden, and used the same method for measuring ADL dependency. However, although the studies were partly conducted during the same time period, the study populations do not overlap, as the Skaraborg Hospitals only joined the one‐year follow‐up in the Swedish stroke register after 2010. We did not replicate the dramatic loss of ADL function during the first year. The explanation for the different findings is not clear, but may partly be attributed to some methodological differences. In the study Ullberg et al. (2015) a relatively large proportion of the population was lost to follow‐up at 12 months, and their analysis was not restricted to first ever stroke. Although highly speculative, it is also possible that local variations in access to rehabilitation and care services may contribute, as we found that deterioration to ADL dependency was associated with a perception of unmet care needs.

The strengths of this study include the long‐term longitudinal follow‐up of a large unselected hospital based cohort with a low portion of participants lost to follow‐up. The study also has some important limitations. The absolute numbers of study participants with ADL dependency at 3 months were low, limiting the statistical power. Moreover, we did not have information about important possible contributors to transitions in ADL status including recurrent strokes during follow‐up, depression and cognitive status. As we used data from the Swedish Stroke Register to measure ADL ability at 3 months, we were limited to use their method for measuring ADL also at the subsequent annual follow‐up. This measure is based on the core variables in well‐established standard scales such as the Barthel Index and the modified Rankin Scale. However, the findings cannot be directly translated to the full scales, which may be considered a limitation. Data for self‐perceived unmet care needs was collected for the first time at follow‐up at 12 months, and not (which would have been ideal) at 3 months, leading to a potential influence of reverse causality on the results, as individuals who deteriorated between 3 to 12 months may be more likely to report their care needs as unmet. However, in sensitivity analyses excluding those with deterioration before 12 months, the association between self‐perceived unmet care needs and deterioration to ADL dependency remained. It should also be noted that patients were recruited 2007–2009. As dependency levels at 3 months have been reported to be decreasing over the last decade, it may be argued that our results are not applicable to more recent cohorts. However, the observed decrease in stroke severity is mainly attributed to better prevention and acute therapy. Here, we investigated the chronic phase with the starting point at 3 months post stroke. For this phase, no new therapies have been introduced over the last decade.

## 5 CONCLUSIONS

In conclusion, in this study we show that despite stable proportions of ADL dependency among stroke survivors at different time points after stroke, transitions between ADL independence and dependency occur up to 5 years after stroke, indicating that the chronic phase after stroke is not static. We also found potentially modifiable factors predicting these transitions, emphasizing the need for interventions and support also during the chronic phase after stroke.

## CONFLICT OF INTEREST

The Authors declare that there is no conflict of interest.

## DATA AVAILABILITY STATEMENT

The data that support the findings of this study are available on request from the corresponding author. The data are not publicly available due to privacy or ethical restrictions.
